# Reduced Infection Efficiency of Phage NCTC 12673 on Non-Motile *Campylobacter jejuni* Strains Is Related to Oxidative Stress

**DOI:** 10.3390/v13101955

**Published:** 2021-09-29

**Authors:** Jessica C. Sacher, Muhammad Afzal Javed, Clay S. Crippen, James Butcher, Annika Flint, Alain Stintzi, Christine M. Szymanski

**Affiliations:** 1 Department of Biological Sciences, University of Alberta, Edmonton, AB T6G 2E9, Canada; jessica.c.sacher@gmail.com (J.C.S.); majavedlh@yahoo.com (M.A.J.); 2 Complex Carbohydrate Research Center, Department of Microbiology, University of Georgia, Athens, GA 30602, USA; clay.crippen25@uga.edu; 3 Ottawa Institute of Systems Biology, University of Ottawa, Ottawa, ON K1H 8M5, Canada; jbutcher@uottawa.ca (J.B.); aflin053@gmail.com (A.F.); astintzi@uottawa.ca (A.S.); 4 Department of Biochemistry, Microbiology and Immunology, Faculty of Medicine, University of Ottawa, Ottawa, ON K1H 8M5, Canada

**Keywords:** *Campylobacter jejuni*, bacteriophage, NCTC 12673, transcriptome, oxidative stress, flagella, glycosylation

## Abstract

*Campylobacter jejuni* is a Gram-negative foodborne pathogen that causes diarrheal disease and is associated with severe post-infectious sequelae. Bacteriophages (phages) are a possible means of reducing *Campylobacter* colonization in poultry to prevent downstream human infections. However, the factors influencing phage-host interactions must be better understood before this strategy can be predictably employed. Most studies have focused on *Campylobacter* phage binding to the host surface, with all phages classified as either capsule- or flagella-specific. Here we describe the characterization of a *C. jejuni* phage that requires functional flagellar glycosylation and motor genes for infection, without needing the flagella for adsorption to the cell surface. Through phage infectivity studies of targeted *C. jejuni* mutants, transcriptomic analysis of phage-resistant mutants, and genotypic and phenotypic analysis of a spontaneous phage variant capable of simultaneously overcoming flagellar gene dependence and sensitivity to oxidative stress, we have uncovered a link between oxidative stress, flagellar motility, and phage infectivity. Taken together, our results underscore the importance of understanding phage-host interactions beyond the cell surface and point to host oxidative stress state as an important and underappreciated consideration for future phage-host interaction studies.

## 1. Introduction

*Campylobacter jejuni* is an important Gram-negative foodborne pathogen worldwide that causes diarrheal disease and is associated with severe post-infectious sequelae, such as Guillain-Barré syndrome, irritable bowel syndrome, growth stunting and arthritis [1,2,3]. Furthermore, reports of antibiotic resistance continue to increase, and thus alternative solutions are required to combat this pathogen [4,5]. Since the most common *Campylobacter* species are commensals in chickens, many *Campylobacter* infections result from mishandling or consumption of contaminated poultry products [1]. As such, strategies aimed at preventing and/or at reducing its colonization of poultry flocks are predicted to have a large impact on reducing the number of human infections [5,6].

Bacteriophages (phages), the viruses that infect bacteria, are highly abundant in all environments harbouring bacteria. Because of their natural ability to specifically recognize and kill their hosts, along with their ability to evolve and adapt alongside their hosts, the exploitation of phages has been considered as a possible antimicrobial strategy [7]. As well, phage-host interaction studies have provided valuable insights into bacterial evolution [8]. Phages have been examined as a means to reduce *Campylobacter* colonization on farms, particularly since phages can be added directly to chicken feed and water sources [5,9]. However, several challenges associated with the broadness of strain coverage and efficiency of phage adsorption and infection must be surmounted before this strategy can be considered feasible in large-scale agricultural settings [9,10,11]. Improved understanding of *Campylobacter* phage-host interactions can directly contribute to the success of this strategy.

*C. jejuni* coats its surface with many glycoconjugates, both protein- and lipid-linked, and these structures largely contribute to strain-strain variability in this species [12,13]. Phages in turn have been shown to encode glycan binding proteins that permit them to interact with a wide variety of bacterial glycans (reviewed in [14]). However, the study of how phages depend on bacterial glycosylation pathways, and the resultant glycans, is still in its infancy. We therefore sought to better understand how *Campylobacter* phages interact with *C. jejuni* glycans. Previous work has shown that *Campylobacter* phages tend to require either capsular polysaccharides (CPS) or flagellar motility for adsorption to host cells [15,16]. Taxonomic classification of *Campylobacter* phages has clustered most CPS-dependent phages into the *Fletchervirus* genus, and most flagellar-dependent phages into the *Firehammervirus* genus [17].

Phage NCTC 12673 is a *Campylobacter jejuni*-specific lytic phage of the *Myoviridae* family of tailed phages [18]. This phage was originally used as a *Campylobacter* typing phage and has been shown to require CPS for *C. jejuni* infection [16]. It is a member of the *Fletchervirus* genus of the *Eucampyvirinae* subfamily, of which most members characterized to date have been shown to be CPS-dependent and flagella-independent [16]. However, the NCTC 12673 phage was also shown not to plaque on the paralyzed flagella *cj0390* (*pflB*) mutant, which expresses immobilized flagella due to a lack of a functional flagellar motor complex [16]. We thus sought to better understand the involvement of flagellar motility in *C. jejuni* infection by NCTC 12673.

Here we describe the observation that phage NCTC 12673 cannot plaque on isogenic mutants of at least four genes (*pseCFGH*) in the *C. jejuni* NCTC 11168 pseudaminic acid biosynthesis pathway for flagellar O-glycosylation, which are also required for filament assembly and thus motility [19,20]. We show that these gene products are not required for phage adsorption, and show using whole transcriptome sequencing that their mutation does not induce expression of anti-phage or stress response pathways. Interestingly, we identified a spontaneous NCTC 12673 phage mutant, MutC, that was able to plaque efficiently on these *pse* mutants and that could also plaque efficiently on *motA* and *motB* mutant strains, which express paralyzed flagella [21]. Additionally, we found that MutC also plaques more efficiently on mutants in the oxidative stress defense response (catalase, Δ*katA* and alkyl-hydroxyperoxidase, Δ*ahpC*) compared to the parent phage, and is less sensitive to oxidative stress caused by the bile salt deoxycholate. This suggests that the reduced plaquing efficiency of phage NCTC 12673 on non-motile strains may be related to the fact that non-motile mutants in *C. jejuni* have increased sensitivity to oxidative stress [22]. These new data support and help further explain our previously reported observations that NCTC 12673 phage infection induces upregulation of oxidative stress defense genes in *C. jejuni*, and that mutation of these genes leads to reduced phage infection efficiency [23]. Lastly, we found that relative to the parent phage, MutC appears to more frequently encode a truncated version of a phase-variable flagellar glycan binding protein, Gp047 (recently renamed FlaGrab, [24]). These results support a model whereby phage expression of FlaGrab during infection may exacerbate oxidative stress in the cell through interaction with flagellar glycan intermediates, suggesting that the phage may have evolved a mechanism of toggling an oxidatively-stressed state in its host, and as a result, its own infection efficiency. Taken together, our results point to a complex interplay between phage infectivity, oxidative stress and the flagellar motility pathway in *C. jejuni*, underscoring the importance of understanding phage-host interactions beyond the cell surface.

## 2. Materials and Methods

### 2.1. Bacterial Growth Conditions

*C. jejuni* NCTC 11168 (MP21) [25] and its isogenic mutants and complement strains were grown on 1.5% NZCYM (Research Products International, Mt Prospect, IL, USA) agar plates, supplemented with 50 µg/mL kanamycin or 12–25 µg/mL chloramphenicol where needed, at 37 °C under microaerobic conditions (85% N_2_, 10% CO_2_, 5% O_2_). *Escherichia coli* strains were grown on LB agar, supplemented with 100 µg/mL ampicillin, 50 µg/mL kanamycin or 25 µg/mL chloramphenicol where needed. The list of bacterial strains and phages used in this study is given in Table 1.

### 2.2. Mutagenesis and Complementation

The mutation of *pseF* in *C. jejuni* 11168 was generated by transferring the mutation from *C. jejuni* 81-176Δ*pseF* (kindly provided by Susan Logan) according to the method described in [29] with some modifications. The *pseF*:*cat* DNA fragment was PCR-amplified from *C. jejuni* 81-176Δ*pseF* using Phusion^®^ high-fidelity DNA polymerase. *C. jejuni* 11168 was transformed with the purified PCR product, transformed colonies were selected on MH agar supplemented with chloramphenicol and successful Δ*pseF* mutants in 11168 were confirmed by PCR.

To generate the *pseH* complementation construct, *pseH* was PCR-amplified using the primer set CS-1158/CS-1034 (5′-GGTAGATCTTTGATAAAACTTAAAAATTTCGCAGA-3′ and 5′-ATACTCGAGTTAGCTAGGCAAGGCTTTGC-3′) and cloned into BglII/XhoI-digested pAJ23 [29]. Plasmids containing the *pseH* construct were PCR-verified and then used to transform *C. jejuni* 11168 Δ*pseH* cells using natural transformation [29]. The complete protocol for natural transformation is available on Protocols.io at https://doi.org/10.17504/protocols.io.magc2bw (accessed on 22 September 2021).

### 2.3. Phage Propagation and Titration

Phage NCTC 12673 and its propagating strain *C. jejuni* 12661 [34] were obtained from the National Collection of Type Cultures (NCTC; Salisbury, UK). Phage MutC was also propagated on *C. jejuni* 12661 following initial isolation on *pseC* cells. Phage propagation and titration were performed following the methods described in [23].

### 2.4. Efficiency of Plating (EOP) Assays

EOP assays were done by spotting serial dilutions of NCTC 12673 phage onto different strains and determining the proportion of plaque forming units formed on mutant strains compared to the corresponding wild type strain, as described in [23]. Briefly, overnight bacterial cultures were harvested in NZCYM broth and set to an OD_600_ of 0.35. A 5-mL aliquot of this suspension was transferred to a standard sized empty Petri dish and incubated at 37 °C without shaking for 4 h under microaerobic conditions. The suspension was then set to an OD_600_ of 0.5, and 200 µL of this was mixed with 5 mL sterile 0.6% molten NZCYM agar at 45 °C. This suspension was poured onto the surface of a pre-warmed NZCYM plate containing 1.5% agar. Plates were allowed to solidify for 15 min and then 10 µL of serial dilutions of a phage suspension (starting at 10^7^ PFU/mL) was spotted onto the agar surface and allowed to completely soak into the agar (15 min) before inverting the plate and incubating at 37 °C under microaerobic conditions. Plaques were counted after 18–24 h and converted to PFU/mL by multiplying countable numbers by the total dilution factor. Percent infectivity was determined by dividing PFU/mL for each test strain by the same value for the wild type reference strain. This method is available on Protocols.io (https://doi.org/10.17504/protocols.io.mahc2b6) (accessed on 22 September 2021).

### 2.5. Deoxycholate Assay

The effect of deoxycholate on the ability of phage to infect *C. jejuni* was tested under conditions previously shown to induce oxidative stress [35] with some modifications. Overnight cultures were harvested and set to OD_600_ of 0.5 in 10 mL of BHI broth in a polystyrene petri dish. Deoxycholate was added to a final concentration of 0.05% and the cultures were incubated for 18 h in microaerobic conditions. After growth in deoxycholate, cultures were collected, washed with fresh broth and the EOP assay was performed as described above.

### 2.6. Adsorption Assays

Adsorption assays were done according to [36] with some modifications. Briefly, cells were harvested as detailed above for growth curves after washing pellets three times in 1 mL NZCYM broth and then set to an OD_600_ of 0.4 in 2 mL NZCYM broth in a small empty Petri plate. Then, 20 μL of 0.22-μm-filtered phage lysate at 5.6 × 10^5^ PFU/mL was added and mixed by pipetting. The number of unadsorbed phages at t = 0 was determined by removing 500 μL immediately after mixing phage and host and centrifuging at 15,000 rpm for 2 min at 4 °C. Then, 100 μL from the supernatant was removed and kept on ice until all samples were ready for titration. After the t = 0 sample was taken, phage-cell suspensions were incubated aerobically with 100 rpm shaking at 37 °C for 90 min, at which point the sampling process was repeated. Samples from each time point were serially diluted in SM buffer and titered for PFU/mL to determine the number of unadsorbed phages over time. Percent adsorption was determined by dividing PFU/mL of unadsorbed phage particles in each test strain by the same value for the wild type reference strain.

### 2.7. Total RNA Extraction

*C. jejuni* 11168 wild type, Δ*pseC* and Δ*pseF* mutant cells were harvested from overnight NZCYM plate cultures, pelleted and washed once in NZCYM broth and set to an OD_600_ of 0.05 (2 × 10^8^ colony forming units per mL (CFU/mL)) in 20 mL NZCYM broth in 125-mL Erlenmeyer flasks, each containing a 1-inch sterile magnetic stir-bar. Cells were grown under microaerobic conditions and magnetically stirred at 200 rpm. After 4.5 h incubation (mid-log phase, cell counts were approximately 5 × 10^8^ CFU/mL), the entire contents of each flask was transferred to a pre-prepared tube containing 2.6 mL (0.1 volume) ice cold 10% buffered phenol in 100% ethanol to stabilize RNA followed by immediate mixing and storage on ice until all samples were collected [37]. RNA was extracted from each sample using a hot phenol method [37]. RNA samples were sequentially DNAse-treated (37 °C for 30 min) using RNAse-free DNAse I (Epicentre, Madison, WI, USA) and cleaned using the Zymo RNA Clean & Concentrator (Zymo Research, Irvine, CA, USA). PCR was used to confirm the absence of residual DNA. Total RNA quality was assessed using an Agilent Bioanalyzer (Agilent Technologies, Santa Clara, CA, USA) and RNA was stored at −80 °C until further use. Samples were extracted in biological triplicate.

### 2.8. RNA-Sequencing

Total mRNA libraries from all replicates were generated. Samples were depleted of rRNA using the RiboZero bacterial kit (Illumina, San Diego, CA, USA) according to the manufacturer’s instructions. Successful rRNA depletion was confirmed using the Agilent Bioanalyzer RNA 6000 Pico Kit (Agilent Technologies, Santa Clara, CA, USA). Strand-specific barcoded sequencing libraries were constructed using the Ion Total RNA-seq kit (Thermo Fisher Scientific, Waltham, MA, USA). Libraries were quality-checked and quantified using the Bioanalyzer High Sensitivity DNA kit (Agilent Technologies, Santa Clara, CA, USA) and pooled together in equimolar amounts. The pooled libraries were templated using the Ion PI Hi-Q kit (Thermo Fisher Scientific, Waltham, MA, USA) and sequenced on an Ion Torrent Proton using the Ion PI Hi-Q sequencing 200 kit (Thermo Fisher Scientific, Waltham, MA, USA) on a single Proton V2 chip.

The raw sequencing reads were demultiplexed by the Ion Torrent suite software (version 5.2.2., Thermo Fisher Scientific, Waltham, MA, USA) and sequentially mapped to the host (NCTC 11168) and phage (NCTC 12673) genomes using Spliced Transcripts Alignment to a Reference (STAR) [38] (Table S1). Reads aligning to coding regions were counted using HT-seq using the default settings. The raw demultiplexed sequencing reads have been deposited at the NCBI Sequence Read Archive (SRA) archive under accession number PRJNA743365. DESeq2 was used to identify differentially expressed transcripts between each strain [39]. Genes with a fold change +/− 1.5 and false discovery rate (FDR)-corrected *p*-value < 0.05 were considered differentially expressed.

### 2.9. Isolation of MutC

MutC was isolated as a clear plaque following an EOP assay (as described above) whereby serial dilutions of phage NCTC 12673 were spotted onto a plate of *C. jejuni* NCTC 11168 *pseC* mutant cells. The plaque was isolated and purified according to the methods of [40] and propagated on *C. jejuni* 12661, the propagating strain for the parent phage, NCTC 12673.

### 2.10. Whole Genome Sequencing and Analysis

Phage DNA (MutC and NCTC 12673) was prepared using hot phenol-chloroform extraction following pre-amplification with phi29 polymerase, as described in [18]. Genomic DNA was submitted for Illumina library preparation and whole-genome sequencing to The Applied Genomics Core (Edmonton, AB, Canada). Genomes of NCTC 12673 (resequenced during this work) and MutC were aligned against the NCTC 12673 reference strain in GenBank (accession: GU296433) [18]. 

Genetic differences between NCTC 12673 and MutC phages, as compared to the reference strain, were identified by variant calling using the Illumina sequencing reads using Geneious (version 8).

## 3. Results

### 3.1. Phage NCTC 12673 Requires a Functional Pseudaminic Acid Biosynthetic Pathway for Infection

**Figure 1:**
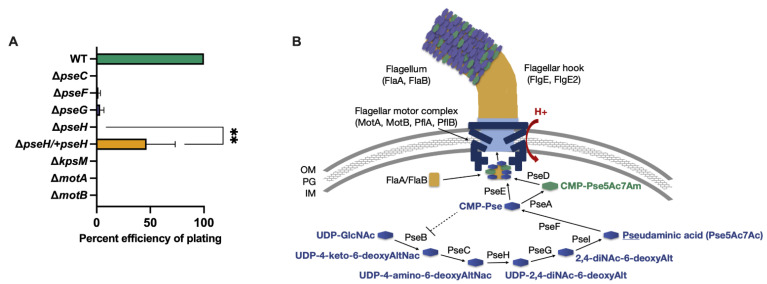
(**A**) Percent efficiency of plating (EOP) of phage NCTC 12673 on *C. jejuni* NCTC 11168 wild type (WT) and isogenic mutants in genes encoding enzymes involved in pseudaminic acid biosynthesis (*pse*), flagellar motor function (*mot*), and *kpsM* (capsular polysaccharide biosynthesis; included as a negative control). Bars represent the mean of three to six biological replicates. One-way ANOVA showed all mutant strains to be statistically significantly different from wild type (*p* < 0.0001). The results of an unpaired *t*-test for Δ*pseH* vs. *pseH* complement strain is indicated by asterisks (*p* = 0.0044). (**B**) Schematic of the pseudaminic acid pathway for flagellar O-glycosylation in *C. jejuni,* which is required for flagellar motility [20]. Dotted line shows PseB is inhibited by CMP-Pse [41]. OM: outer membrane, PG: peptidoglycan, IM: inner membrane, Pse5Ac7Ac: pseudaminic acid, Pse5Ac7Am: acetamidino-modified pseudaminic acid.

To determine whether flagellar glycosylation is important for NCTC 12673 infection, we examined phage infection of Δ*pseC*, Δ*pseF*, Δ*pseG*, Δ*pseH* mutants and found that compared to wild type *C. jejuni* NCTC 11168 infection (EOP set to 100%), infectivity was decreased on all mutants (Figure 1). No plaquing was observed on Δ*pseC* or Δ*pseH*, and infectivity relative to wild type was drastically reduced upon infection of *pseF* (EOP = 1.7%; *p* = 0.0001) and *pseG* (EOP = 3.3%; *p* = 0.0001). We complemented Δ*pseH* with a functional copy of the gene and found that infectivity was significantly improved in the Δ*pseH/+pseH* complemented strain (EOP = 46.6%; *p* = 0.0044) compared to the *pseH* mutant. As a negative control, we tested infectivity on *kpsM*, which lacks the capsule required for NCTC 12673 binding and is known not to be infected by the NCTC 12673 phage [16].

### 3.2. Phage NCTC 12673 Adsorbs to Pseudaminic Acid Pathway Mutants at Wild Type Levels

**Figure 2:**
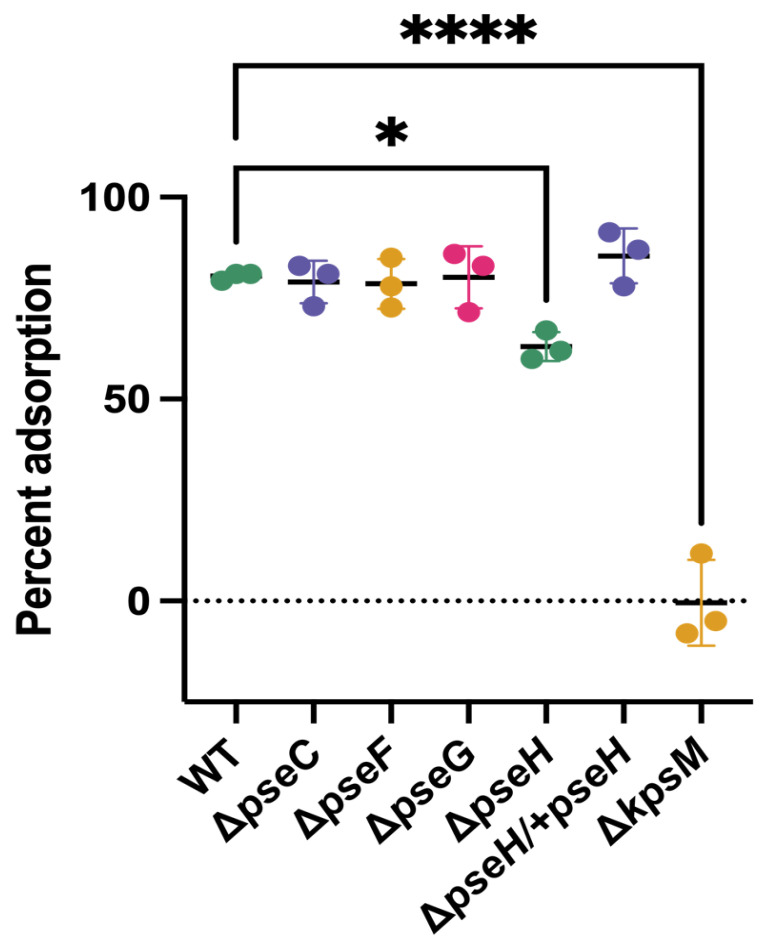
Percent phage NCTC 12673 adsorption to *C. jejuni* NCTC 11168 wild type and Δ*pseC*, Δ*pseF* Δ*pseG*, Δ*pseH*, Δ*pseH*/ + *pseH*, and Δ*kpsM* mutants. Bars represent the mean of three biological replicates, and error bars represent standard deviations. Statistical significance as determined by one-way ANOVA is indicated by asterisks as follows: *p* < 0.05 (*) and *p* < 0.0001 (****). All other mutant strains were shown to have no significant difference from wild type.

It has been well established that inactivation of *pseB, pseC, pseE, pseF, pseG, pseH* or *pseI*, which all encode enzymes required for biosynthesis and transfer of pseudaminic acid to flagella in *C. jejuni*, prevents flagellar biogenesis and motility [20,42] (Figure S1). As other *Campylobacter* phages have been shown to depend on flagellar motility for infection, and this has been attributed to the use of flagella as a surface receptor for the phage [36], we first sought to determine whether the reason NCTC 12673 infectivity was impaired on *pse* mutants was due to a lack of adsorption to these aflagellate mutants. We titered the number of free phages remaining in the supernatant following incubation with *C. jejuni* NCTC 11168 wild type, Δ*pseC, ΔpseF*, Δ*pseG* and Δ*pseH* mutants, a Δ*pseH*/ + *pseH* complement strain, and a Δ*kpsM* mutant as a negative control. With the exception of Δ*pseH*, which showed a slight, yet statistically significant decrease in adsorption compared to wild type cells (*p* = 0.002), we observed no differences in adsorption for most mutant strains relative to wild type cells (Figure 2). In line with previous results, NCTC 12673 phage did not adsorb to Δ*kpsM* mutant cells (*p* = 0.0002).

### 3.3. ΔpseC and ΔpseF Mutants Display No Evidence of Stress Response or Phage Defense, but Downregulate Many Flagellar Genes

**Figure 3:**
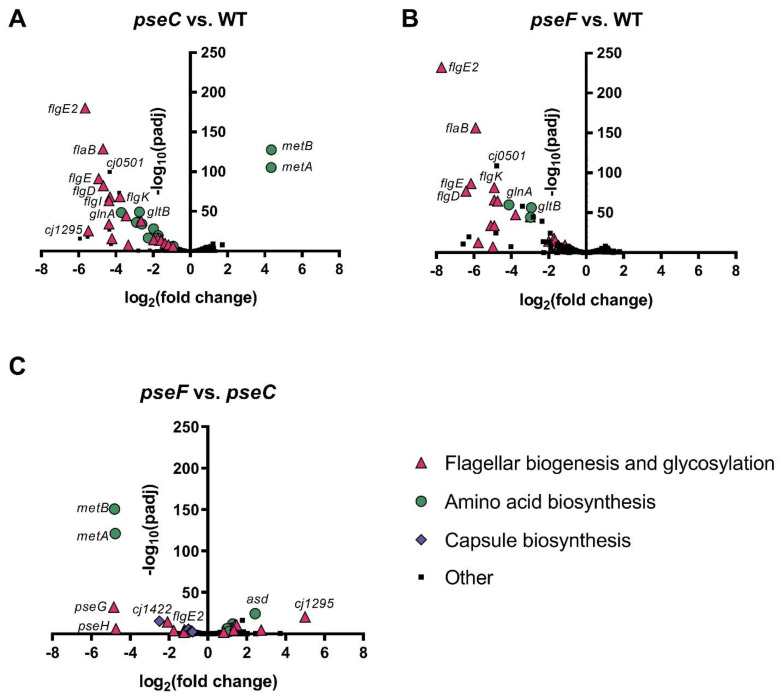
Volcano plots showing the most significantly differentially expressed genes between *C. jejuni* NCTC 11168 wild type, Δ*pseC* and Δ*pseF* cells. Significantly differentially expressed genes between Δ*pseC* and wild type (**A**), between Δ*pseF* and wild type (**B**) and between Δ*pseF* and Δ*pseC* (**C**) are shown. The negative log of the false discovery rate (FDR)-adjusted *p*-value vs. the log_2_ fold change between the conditions indicated is plotted for each gene. Genes with a fold change +/− 1.5 and FDR-corrected *p*-value < 0.05 were considered differentially expressed. Selected significantly differentially expressed genes are represented by different colours and symbols according to their predicted or known function.

We next sought to determine whether downstream effects of *pse* mutation led to stress responses or expression of any anti-phage defense genes, which might help explain why the NCTC 12673 phage is unable to efficiently infect these strains. To test this, we used RNA-seq to compare gene expression between *C. jejuni* NCTC 11168 wild type, Δ*pseC* and Δ*pseF* mutant cells during the mid-log phase of growth (Figure S2). We identified widespread downregulation of many genes in both Δ*pseC* compared to wild type cells (Figure 3A, Table S1) and Δ*pseF* compared to wild type cells (Figure 3B, Table S1). For both mutant strains relative to wild type cells, most of the significantly downregulated genes were related to flagellar biogenesis (e.g., *flgE2, flaB, flgE,* *flgD,* *flgI, flgK*), with the next most altered category being amino acid biosynthesis (e.g., *gltB, glnA*). *cj0501*, a pseudogene [43], was downregulated in Δ*pseC* and Δ*pseF*. Interestingly, relative to wild type and Δ*pseF,* the Δ*pseC* mutant showed substantial upregulation of *metA* and *metB* genes (encoding enzymes for methionine biosynthesis). We also observed the slight upregulation of the CPS biosynthesis genes (*cj1422c, hddA*) in Δ*pseC* mutant cells compared to wild type cells. Direct comparison of Δ*pseF* to Δ*pseC* showed limited differences but did highlight that *metB* and *metA* were downregulated in Δ*pseF* cells compared to Δ*pseC* cells, as were *pseG*, *pseH*, and *cj1422c*, while *asd*, *cj1295*, and *cj1022c* were expressed more highly in Δ*pseF* than Δ*pseC* (Figure 3C, Table S1).

### 3.4. Phage NCTC 12673 Is Unable to Infect Cells in the Absence of the Flagellar Motor Proteins MotA or MotB

To better understand the possible NCTC 12673 dependence on flagellar motility, and to verify previous results that showed that this phage did not plaque on a “paralyzed” (full-length but non-motile) flagella Δ*pflB* mutant, we tested NCTC 12673 phage plaquing efficiency on flagellar motor mutants Δ*motA* and Δ*motB* [21,24]. Interestingly, we found that NCTC 12673 phage infection was essentially abolished on both mutants (Figure 1A).

### 3.5. Oxidative Stress Sensitivity of Non-Motile Mutant Strains May Explain Reduced NCTC 12673 Plaquing Efficiency

We recently showed that the NCTC 12673 phage displays reduced plaquing on mutants in the oxidative stress defense genes Δ*katA*, Δ*ahpC* and Δ*sodB* [23]. As well, Flint et al. previously showed that many non-motile *C. jejuni* NCTC 11168 mutants, including Δ*flgK*, Δ*flgH*, Δ*flgD*, Δ*flgI*, Δ*flgR*, Δ*pseB*, and Δ*motAB*, are hypersensitive to oxidative stress, presumably through disruption of the proton gradient/electron leakage through the electron transport chain increasing reactive oxygen species [22]. With the exception of Δ*motA* and Δ*motB,* we observed that all of these genes highlighted to lead to oxidative stress sensitivity by Flint et al. were among the most significantly downregulated genes across the transcriptome in both Δ*pseC* and Δ*pseF* mutant strains (Figure 3A,B). We therefore hypothesized that the observed dependence of NCTC 12673 on flagellar motility might be linked to increased oxidative stress sensitivity in these strains.

### 3.6. A spontaneous NCTC 12673 Mutant Phage, “MutC”, Efficiently Plaques on Both Non-Motile and Oxidative Stress Defense Mutants

**Figure 4:**
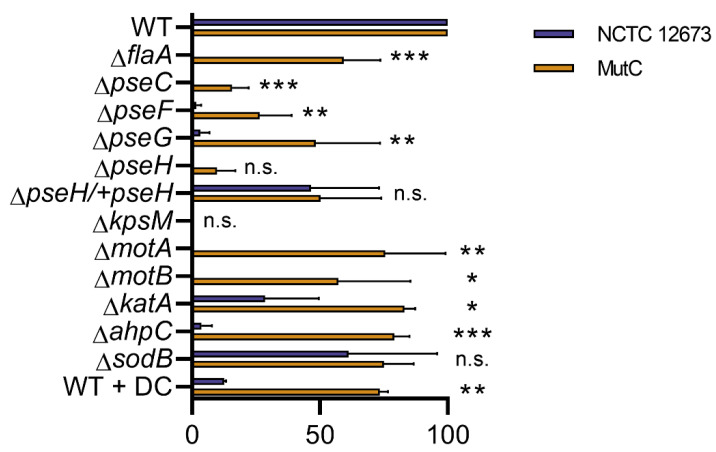
Percent efficiency of plating (EOP) of phages NCTC 12673 and MutC on *C. jejuni* NCTC 11168 wild type (WT) and isogenic mutants in pseudaminic acid biosynthesis (*pseCFGH*), flagellar motor (*motAB*), and oxidative stress defense genes (*katA, ahpC, sodB*), as well as WT cells in the presence of 0.05% deoxycholate (DC), an oxidative stress-inducing agent for *Campylobacter* [35]. Bars represent the mean of three to six biological replicates (with the exception of WT+DC, which represents the mean of two biological replicates). Statistical significance between NCTC 12673 and MutC phage for each strain was determined by unpaired *t*-test and is indicated by asterisks as follows: *p* < 0.05 (*), *p* < 0.01 (**), *p* < 0.001 (***). n.s: no significant difference between NCTC 12673 and MutC.

To better understand the mechanism for motility dependence by NCTC 12673, we sought to characterize an NCTC 12673 phage escape mutant, MutC, which we isolated as a clear plaque on Δ*pseC* mutant cells. Interestingly, we found that MutC displayed a significantly higher EOP on all of the Δ*pse* and Δ*mot* mutants tested compared to the parent phage, NCTC 12673 (Figure 4). While the parent phage displayed undetectable plaquing on Δ*pseC*, Δ*pseH*, Δ*motA*, and Δ*motB*, and low efficiency on Δ*pseF* and Δ*pseG* (Figure 4), phage MutC plaqued at a statistically significantly higher efficiency than the parent phage on Δ*pseC* (15.7%; *p* = 0.001), Δ*pseF* (26.4%; *p* = 0.002), Δ*pseG* (48.4%; *p* = 0.003), Δ*pseH* (9.8%; *p* = 0.01), Δ*motA* (75.6%; *p* = 0.005), and Δ*motB* (57.3%; *p* = 0.02), as determined by *t*-test. Similar to the parent phage, MutC did not infect Δ*kpsM* cells, suggesting that it retained dependence on *C. jejuni* CPS.

To determine whether the ability to overcome dependence on motility by MutC also allowed this phage to overcome dependence on oxidative stress defense genes, we next tested MutC infection of mutants in the oxidative stress defense genes Δ*katA* (catalase), Δ*ahpC* (alkyl-hydroxyperoxide reductase), and Δ*sodB* (superoxide dismutase) [31]. We found that MutC more efficiently infected Δ*katA* (83.0% vs. 28.6%; *p* = 0.01) and Δ*ahpC* (79.2% vs. 3.7%; *p* = 0.00005) mutant cells compared to phage NCTC 12673 (Figure 4). Conversely, we did not observe a difference between MutC and NCTC 12673 infection of the Δ*sodB* mutant (75.0% vs. 61.2%; *p =* 0.62).

### 3.7. Phage MutC Is Less Impacted by Exposure to the Oxidative Stress-Inducing Agent Deoxycholate Than Phage NCTC 12673

To further demonstrate that phage MutC had gained an improved ability to infect oxidatively-stressed cells, we compared MutC and the parent phage NCTC 12673 infection efficiency on *C. jejuni* 11168 following exposure to 0.05% deoxycholate, a bile salt previously shown to induce the production of intracellular reactive oxygen species in *C. jejuni* when supplemented in the growth medium [35]. We found that MutC indeed had a significantly improved (5.8-fold) infection efficiency following growth in the presence of deoxycholate compared to NCTC 12673 (73.5% vs. 12.6%, respectively; *p* = 0.001) (Figure 4).

### 3.8. Genomic Comparison between Phages NCTC 12673 and MutC Predicts Differences in Several Proteins, including FlaGrab, a Flagellar Glycan-Binding Protein

In order to understand the mechanism for the observed differences in NCTC 12673 and MutC plaquing on non-motile and Δ*katA*/Δ*ahpC* mutants, we performed whole-genome sequencing of both phages and assessed differences in their genomes. Although the two phages displayed nucleotide changes at approximately a dozen locations across their 135-kbp genomes (Table S2), amino acid-level consequences were predicted in only six genes: *gp047*/*flagrab*, which encodes the flagellar glycan-binding protein FlaGrab [24,29,30]; *gp041,* annotated as a baseplate wedge protein homologue; *gp058* and *gp167,* both annotated as homing endonucleases; and *gp114* and *gp116,* both hypothetical protein-encoding genes (Table 2). We chose to focus on the *flagrab* variation for follow-up analysis since our previous studies documented associations between FlaGrab and the *C. jejuni* flagellar motility pathway, leading us to surmise that it might be implicated in the NCTC 12673/MutC flagellar mutant infectivity differences observed.

### 3.9. Differences in Variant Frequency within Two Poly-Adenosine Nucleotide Tracts Suggests MutC Phage Expresses a Truncated FlaGrab Protein More Frequently Than NCTC 12673 Phage

FlaGrab (encoded by *gp047*/*flagrab*) is a flagellar glycan binding protein encoded by all *Campylobacter* phages characterized to date [17], which we have previously shown binds to acetamidino-modified pseudaminic acid glycans on *C. jejuni* flagella [29], inhibits *C. jejuni* growth [24,30] and reduces expression of energy metabolism genes following binding to motile flagella [24]. Within the NCTC 12673 phage genome, we identified the presence of two poly-A tracts within *flagrab.* Variation in the length of either or both poly-A tracts is predicted to lead to frameshift mutations that would severely truncate the protein; when both tracts contain 7 As, the predicted FlaGrab protein is 1365 amino acids in length, whereas encoding 6 As at one or both tracts results in a protein of less than or equal to 83 amino acids. By aligning the reads generated by short-read whole genome sequencing (Illumina) data for MutC and NCTC 12673 phages to the NCTC 12673 reference genome in GenBank (accession: GU296433) [18], we analyzed the length of each of the two poly-A tracts within the *flagrab* gene for both phages and compared the frequency of 6-A vs. 7-A tracts to determine whether we would expect differences in FlaGrab length expressed by the two phages (Table 2). At the first poly-A tract (chromosomal position: 40,821), we observed that MutC encoded the 6-A allele at a frequency of 68.80%, whereas NCTC 12673 did not show variability, encoding only the 7-A allele. At the second tract (chromosomal position: 40,935), we observed the 6-A allele in similar proportions for both phages (NCTC 12673: 33.20%, MutC: 25.30%). These data suggest that while both phages would be predicted to express a truncated FlaGrab as a result of variation at the second tract approximately one third of the time, phage MutC is predicted to express a truncated FlaGrab most of the time, and thus far more frequently than NCTC 12673, due to variation at both tracts.

To verify the *flagrab* variability observed upon whole-genome sequencing, we designed primers to PCR-amplify the poly-A tract regions of *flagrab.* We then performed Sanger sequencing on the resultant amplicons using NCTC 12673 and MutC phage lysates of *C. jejuni* NCTC 12661 cells (the strain routinely used to propagate these phages) as templates. Indeed, we found variability in sequence data (represented by multiple chromatogram peaks) starting directly downstream of the *flagrab* poly-A tracts for both phages, supporting the variability predicted from the variant frequency analysis described above.

## 4. Discussion

Phage NCTC 12673 is classified as a member of the *Fletchervirus* genus of *Campylobacter* phages, members of which tend to target CPS and not flagella [15,17,18]. Coward et al. (2006) screened this phage against a *C. jejuni* mutant library and showed that acapsular mutants were consistently resistant to the phage [16], and we have confirmed that acapsular mutants are not infected or bound by the phage [29] (this work). However, Coward et al. also found that there was one non-motile mutant that NCTC 12673 could not infect: the paralyzed flagellar mutant Δ*pflB* [16]. This observation suggested that in addition to dependence on CPS for infection, motile flagella may also be important for successful infection of *C. jejuni* cells by phage NCTC 12673.

We sought to elucidate whether host cell motility was indeed important for NCTC 12673 infection, and if so, why. We analyzed whether phage NCTC 12673 could plaque on several mutants of the pseudaminic acid (*pse*) flagellar glycosylation pathway, which are known to be aflagellate and non-motile [44] (Figure S1). We found that mutagenesis of genes in the *pse* pathway (*pseC, pseF, pseH,* or *pseG)* drastically reduced NCTC 12673 phage ability to infect *C. jejuni* NCTC 11168 cells, and that complementation of *pseH in trans* restored flagellar motility and improved phage infection efficiency. These results supported our hypothesis that NCTC 12673 phage may indeed have a dependency on *C. jejuni* flagellar motility.

One of the simplest explanations for why a phage might require flagellar motility is that it uses motile flagella as a host cell surface receptor and binds to the flagella as part of its initial interaction with the host; indeed, others have shown that this is the case for the flagella-requiring *Campylobacter* phage F341 [36]. To determine if this was the case for NCTC 12673, we assessed adsorption of the NCTC 12673 phage to our set of *pse* mutants. Surprisingly, we found that the phage could still adsorb to the *pse* mutants at wild type levels, ruling this out as an explanation. We thus hypothesized that *pse* mutation may impact phage infection at a later point in the phage lifecycle such as during the intracellular phase of phage infection. We thus sought to determine whether we could detect any other impacts of *pse* mutations on *C. jejuni* cells that might help explain why the phage could not infect these mutants, such as host stress responses or anti-phage defense that might be detrimental to phage replication, or other unexpected downstream effects of *pse* mutagenesis.

Since downstream effects on gene expression in response to gene mutagenesis are common, we were interested in understanding the total effects of *pse* gene inactivation to identify possible explanations for the lack of phage infectivity of *pse* mutants. To test this, we performed RNA-seq on Δ*pseC* and Δ*pseF* mutant cells. We did not observe signs of general stress response activation in the Δ*pseC* and Δ*pseF* mutants relative to wild type cells, or increased expression of any known anti-phage defense genes [23,45], but we did observe widespread downregulation of many flagellar genes. This downregulation was not surprising, since *C. jejuni* is known to tightly regulate flagellar expression at multiple levels including hierarchical expression of genes through distinct sigma factors (σ70, σ54, σ28), feedback inhibition by σ28 expression, global transcriptional regulation by CsrA-FliW [46,47], PseB inhibition by Pse glycans and *pse* gene requirement for filament biogenesis [41,48,49,50,51]. It is therefore expected that the cell would downregulate σ54- and σ28-regulated flagellar biogenesis genes in response to the lack of σ28-regulated *pse* gene expression, which is what we observed.

Interestingly, Flint et al. (2014) showed that several non-motile *C. jejuni* mutants in genes downregulated in our Δ*pseC* and Δ*pseF* mutants are hypersensitive to oxidative stress [22]. Additionally, our recent transcriptomic analysis of NCTC 12673 phage during lytic infection of *C. jejuni* 11168 showed that upon infection, *C. jejuni* upregulates the oxidative stress defense gene *katA* as well as the heme acquisition genes *chuABCDZ,* which import the heme co-factor required for KatA activity [23]. This led to the finding that Δ*katA,* as well as Δ*ahpC* and Δ*sodB*, which constitute other oxidative stress response genes, are important for efficient phage infection by NCTC 12673, suggesting that this phage cannot efficiently infect oxidatively-stressed cells [23]. In support of this, Chatterjee et al. (2020) have recently shown that oxidative stress response genes are also important for *Enterococcus faecalis* phage infection, suggesting that oxidative stress may play an important role in dictating phage-host dynamics more broadly [52]. We therefore hypothesized that increased sensitivity to oxidative stress of *pse* mutants, brought on by their lack of motility, might explain the inability of NCTC 12673 phage to plaque on the *pse* mutants.

During our experiments plaquing phage NCTC 12673 on the *pseC* mutant, we isolated a spontaneous phage variant that could form clear plaques on Δ*pseC* mutant cells. We decided to characterize this variant, which we named MutC, to see if it might provide clues into why *pse* mutation impacted NCTC 12673 infection efficiency. We tested MutC’s ability to plaque on the other *pse* mutants and found that in addition to plaquing on Δ*pseC* cells, MutC could efficiently infect mutants in Δ*pseC*, Δ*pseF*, Δ*pseG* and Δ*pseH*, as well as the flagellar filament mutant Δ*flaA* and the paralyzed flagellar mutants Δ*motA* and Δ*motB*.

To validate the hypothesis that increased oxidative stress was the reason the *pse* and *mot* mutants were not efficiently infected by phage NCTC 12673, we next sought to determine whether MutC could plaque on *C. jejuni* mutants lacking oxidative stress defense genes. Interestingly, we found that MutC was able to plaque more efficiently than the parent phage on the oxidative stress defense mutants Δ*katA* and Δ*ahpC.* In other words, the mutation(s) in MutC that allowed it to overcome dependence on *pse* genes also allowed it to overcome dependence on oxidative stress defense genes, providing a clear link between *pse* mutation and oxidative stress defense. This result supported our hypothesis that NCTC 12673′s inability to infect *pse* and *mot* mutants may be caused by oxidative stress sensitivity of these mutants. Of note, NCTC 12673 and MutC similarly infected a Δ*sodB* mutant, suggesting that the phages may be sensitive to specific oxidative stresses, as hydrogen peroxides/alkyl peroxides are detoxified by KatA/AhpC while superoxides are detoxified by SodB [31]. Alternatively, this could indicate the involvement of other regulators known to impact oxidative stress regulation, such as the *Campylobacter* oxidative stress regulator, CosR [53].

To further demonstrate that phage MutC had gained an improved ability to infect oxidatively-stressed cells, we compared phage infection efficiency on *C. jejuni* 11168 of MutC and the parent phage NCTC 12673 following exposure to 0.05% deoxycholate, a bile salt known to induce the production of reactive oxygen species in *C. jejuni* [35]. We found that MutC indeed had 5.8-fold higher infection efficiency following deoxycholate exposure compared to NCTC 12673. This provided further support to the hypothesis that the sensitivity of phage NCTC 12673 to oxidative stress explains its apparent dependence on a functional flagellar pathway.

Reanalyzing our transcriptomic data given these new findings about oxidative stress sensitivity of the *pse* mutants, we found it noteworthy that the Δ*pseC* mutant displayed higher expression of the methionine biosynthesis genes *metA* and *metB* compared with wild type or the Δ*pseF* mutant. Others have proposed that increased methionine biosynthesis may be necessary to regenerate oxidized methionines that exceed the capacity of the methionine sulfoxide reductase repair system, and/or for the increased production of oxidative repair enzymes such as KatA and AhpC, which are known for their high methionine content/quenching abilities in the related pathogen *Helicobacter pylori* [54,55]. It should also be noted that *C. jejuni* does not encode genes for the production of glutathione [56], a key redox buffer involved in the repair of oxidized methionine/cysteine [57]. Of note, our results suggest that mutation of early *pse* pathway genes (*pseC*, *pseH*) is more detrimental to phage infection compared to mutation of late *pse* pathway genes (*pseG*, *pseF*), but the reason for this remains unknown. It is possible that differences in *metA*, *metB*, CPS biosynthesis and/or amino acid biosynthesis may explain some of the difference between NCTC 12673 infectivity of these strains.

To understand the molecular mechanism driving the observed differences in host requirements between the two phages, we sought to identify the genetic differences between NCTC 12673 and MutC. While we identified several genes with variations between the two phages, only five showed amino acid-level changes. Interestingly, one of the amino acid-level differences between the two phages was in FlaGrab*,* a conserved *Campylobacter* phage protein that we previously showed is a flagellar glycan-binding protein specific for acetamidino-modified pseudaminic acid [29] that causes growth inhibition in *C. jejuni*, presumably also through disruption of the PMF leading to oxidative stress [24]. We chose to focus on the FlaGrab variation for follow-up analysis, as its documented association with the *C. jejuni* flagellar motility pathway led us to surmise that it might be implicated in the NCTC 12673/MutC flagellar mutant infectivity differences observed.

Interestingly, the difference we observed in the *flagrab* gene between the two phages was in the frequency of variation at a 6–7-nucleotide poly-A tract, predicted to lead to a frameshift mutation in *flagrab* more frequently in MutC than in NCTC 12673. In fact, phase variability through hypermutable poly-G tracts (a rapid adaptation mechanism that provides a stochastic on/off switch for gene expression via homopolymeric nucleotide stretches encoded within open reading frames [58]) has recently been reported in *Campylobacter* phages [59]. As *C. jejuni* expresses at least 30 phase-variable genes, many of them governing surface structure expression [26,58,60], it is not surprising to find that *Campylobacter* phages have evolved similar mechanisms to ensure evolutionary dominance over such a variable host. Tracts of only 6–7 nucleotides in length would not be predicted to switch on and off as frequently as longer tracts, given the slipped-strand mispairing mechanism that leads to variation at these loci, but would still be reasonably expected to represent a mechanism of variation that could provide an adaptive advantage for the encoding biological entity. The differences in variant frequency at the poly-A tracts in *flagrab* that we observed suggest that a much larger proportion of the MutC population expresses a severely truncated (or “off-switched”) version of FlaGrab compared to the parent phage NCTC 12673. Although we have extensively described the effects of adding recombinantly purified FlaGrab to *C. jejuni* cells, namely that binding of this protein to *C. jejuni* flagella leads to growth inhibition [24,30], the function of FlaGrab in the phage life cycle has remained elusive. And yet, every *Campylobacter* phage characterized to date has been shown to encode a FlaGrab homologue, suggesting it is of high importance to any phage infecting this species [17]. We are still exploring how FlaGrab may or may not be involved in the phenotypes observed, but based on our results to date, we hypothesize that full-length FlaGrab may prevent NCTC 12673 phage from lysing its host under conditions of oxidative stress, while MutC, encoding a truncated FlaGrab, is able to constitutively lyse its host. How this occurs is currently purely speculative, but our hypothesis is that FlaGrab may have another function intracellularly, as many other phage proteins have been shown to have [61]. Our future directions will seek to establish whether a definitive link can be made between the length of FlaGrab and phage infection efficiency.

Although most *Campylobacter* phages studied to date are lytic and not lysogenic, a ‘carrier state’ association between two NCTC 12673-related phages and *C. jejuni* has been described [62]. This is defined as a longer-term association between the phage and host, where the phage associates with the host both intracellularly (episomally) and extracellularly (by associating with the surface) and waits to lyse the cell for a period of time, without integrating into the genome. The carrier state in *C. jejuni* has been shown to lead to downregulation of flagellar motility, suggesting that motility and the phage life cycle may be linked for phages targeting this organism [62]. However, the mechanism for how *C. jejuni* phages might accomplish this has not been elucidated. Given our results here, which point toward FlaGrab having a role in blocking the lytic phage life cycle under non-motile conditions, it is tempting to speculate that the lack of phage infection efficiency we have described here is in fact representative of the carrier state, with FlaGrab representing the switch that dictates the lysis/no-lysis decision.

Together our data suggest a possible role for *flagrab* in dictating NCTC 12673 phage interactions with oxidatively stressed *C. jejuni* cells. However, the data do not rule out possible contributions of the other observed genetic changes between the two phage variants, and the effects of these changes should also be examined. Our preliminary work suggests the L to S amino acid change in MutC *gp041*, predicted to encode a baseplate wedge subunit protein, is reproducible (could be confirmed by Sanger sequencing of phage genomic DNA), suggesting that this change could also play a role in the phenotypic differences between the phages. In addition, analysis of *gp114* and *gp116* has shown that these genes exhibit phase-variability via poly-nucleotide tracts. However, we found these tracts to be highly variable following both Δ*pseC* and wild type infection for both NCTC 12673 and MutC phages, thus reducing the likelihood that on/off switching in these genes is responsible for the stable difference in phenotypes observed between the two phages. Finally, our efforts to confirm the observed differences in the homing endonucleases *gp058* and *gp167* between NCTC 12673 and MutC phages showed that the nucleotide changes observed upon whole genome sequencing were not reproducible, suggesting against a role for these changes in explaining the phenotypic differences between NCTC 12673 and MutC.

## 5. Conclusions

Overall, this work provides novel insights into NCTC 12673 phage interactions with *C. jejuni* 11168. We have shown that the apparent dependence of phage NCTC 12673 on *C. jejuni* motility is not related to adsorption to the flagella, but instead can be explained by the phage’s reduced infection efficiency under conditions of oxidative stress, previously shown to be the case for non-motile *C. jejuni* mutants. We have also shown evidence that phage-encoded FlaGrab, a flagellar glycan binding protein encoded by all *Campylobacter* phages with an unknown role in the phage life cycle, is phase-variable, and that variable expression of this protein may be linked to a mechanism by which the phage can tune its infectivity according to the oxidative stress state of the cell. Taken together, our results point to a complex interplay between phage infectivity, oxidative stress and flagellar motility in *C. jejuni*, underscoring the importance of understanding phage-host interactions beyond the cell surface.

## Figures and Tables

**Table 1:** List of strains and phages used in this study.

Strain	Description/Phenotype	Reference
*C. jejuni* NCTC 11168	Human enteropathy isolate, capsular, motile	[26]
*C. jejuni* NCTC 11168∆*motA*	Non-motile (paralyzed flagella)	[24]
*C. jejuni* NCTC 11168∆*motB*	Non-motile (paralyzed flagella)	[24]
*C. jejuni* NCTC 11168∆*kpsM*	Acapsular	[27]
*C. jejuni* NCTC 11168∆*pseC*	Non-motile (aflagellate)	[28]
*C. jejuni* NCTC 11168∆*pseH*	Non-motile (aflagellate)	[29]
*C. jejuni* NCTC 11168∆*pseH/+pseH*	Motile	This work
*C. jejuni* NCTC 11168∆*pseG*	Non-motile (aflagellate)	[30]
*C. jejuni* NCTC 11168∆*pseF*	Non-motile (aflagellate)	This work
*C. jejuni* NCTC 11168∆*katA*	Hypersensitive to oxidative stress (lacks catalase)	[31]
*C. jejuni* NCTC 11168∆*ahpC*	Hypersensitive to oxidative stress (lacks alkyl hydroxyperoxide reductase)	[31]
*C. jejuni* NCTC 11168∆*sodB*	Hypersensitive to oxidative stress (lacks superoxide dismutase)	[31]
*C. jejuni* NCTC 11168∆*flaA*	Non-motile (aflagellate)	[32]
NCTC 12673	UK phage typing scheme phage 1	[18,33]
MutC	Spontaneous variant of NCTC 12673	This work

**Table 2:** Variant frequency table showing amino acid-level genetic differences between NCTC 12673 and MutC phages. For each gene displaying one or more variants between the two phages, the variants are listed, along with nucleotide change(s) relative to the NCTC 12673 reference strain in GenBank (accession: GU296433) [18], the corresponding amino acid change, the variant frequency, and the corresponding phage genome for each. (A)7 → (A)6: AAAAAAA → AAAAAA. *gp114*/*gp115*: change observed in the region where these genes overlap.

Gene.	Predicted Function	Variant	Phage	Position within Genome	Nucleotide Change	Amino Acid Change	Variant Frequency
*gp041*	Gp6 baseplate wedge subunit	1	MutC	31,844	A → G	L → S	72.70%
		2	MutC	31,885	C → T	M → I	25.60%
*gp047*/*flagrab*	FlaGrab, flagellar glycan binding protein	1	MutC	40,821	(A)7 → (A)6	Frame Shift	68.80%
		2	NCTC 12673	40,935	(A)7 → (A)6	Frame Shift	33.20%
			MutC	40,935	(A)7 → (A)6	Frame Shift	25.30%
*gp058*	Hef59 homing endonuclease	1	MutC	50,606	AA → GG	E → G	69.8%
*gp114*/*gp115*	Hypothetical protein	1	MutC	100,737	(CC)4 → (CC)5	Frame Shift	37.00%
			NCTC 12673	100,737	(C)9 → (C)10	Frame Shift	31.20%
			MutC	100,737	(C)9 → (C)10	Frame Shift	43.70%
*gp116*	Hypothetical protein	1	NCTC 12673	101,692	(C)11 → (C)12	Frame Shift	28.90%
		2	NCTC 12673	101,691	(C)11 → (C)10	Frame Shift	27.10%
			MutC	101,691	(C)11 → (C)10	Frame Shift	62.80%
		3	MutC	101,202	G → A	T → M	68.70%
		4	NCTC 12673	101,112	G → T	T → N	73.80%
*gp167*	Hef168 homing endonuclease	1	MutC	132,316	C → T	D → N	64.00%

## Data Availability

The raw demultiplexed RNA-sequencing reads have been deposited at the NCBI Sequence Read Archive (SRA) archive under accession number PRJNA743365.

## Supplementary Materials

The following are available online at https://www.mdpi.com/article/10.3390/v13101955/s1, Figure S1: Transmission electron micrographs showing presence of flagella in wild type *C. jejuni* 11168 cells (**A**), absence of flagella in *pseC-H* mutants (**B**–**E**), and restoration of flagella in the *pseH*/+*pseH* complement strain. Figure S2: Principal Component Analysis plot for *C. jejuni* NCTC 11168 wild type and mutant cells: wild type, Δ*pseC*, and Δ*pseF*. Table S1: Differentially expressed genes for *C. jejuni* NCTC 11168 wild type and mutant cells: wild type, Δ*pseC*, and Δ*pseF*. Table S2: Complete list of genetic polymorphisms and variant frequencies between NCTC 12673 phage (parent) and MutC, a spontaneous mutant phage isolated on *C. jejuni* 11168 Δ*pseC* mutant cells.
