# Supplementary material for: Reduced Infection Efficiency of Phage NCTC 12673 on Non-Motile Campylobacter jejuni Strains Is Related to Oxidative Stress
Source: Viruses. 2021 Sep 29;13(10):1955. doi: 10.3390/v13101955 (PMC8540345; doi:10.3390/v13101955)
Supplement: Supplementary file 1 [file viruses-13-01955-s001.zip › viruses-1308786-supplementary.pdf]

Supplementary data for Sacher et al. manuscript:

Reduced infection efficiency of phage NCTC 12673 on non-motile *Campybacter jejuni* strains is related to oxidative stress

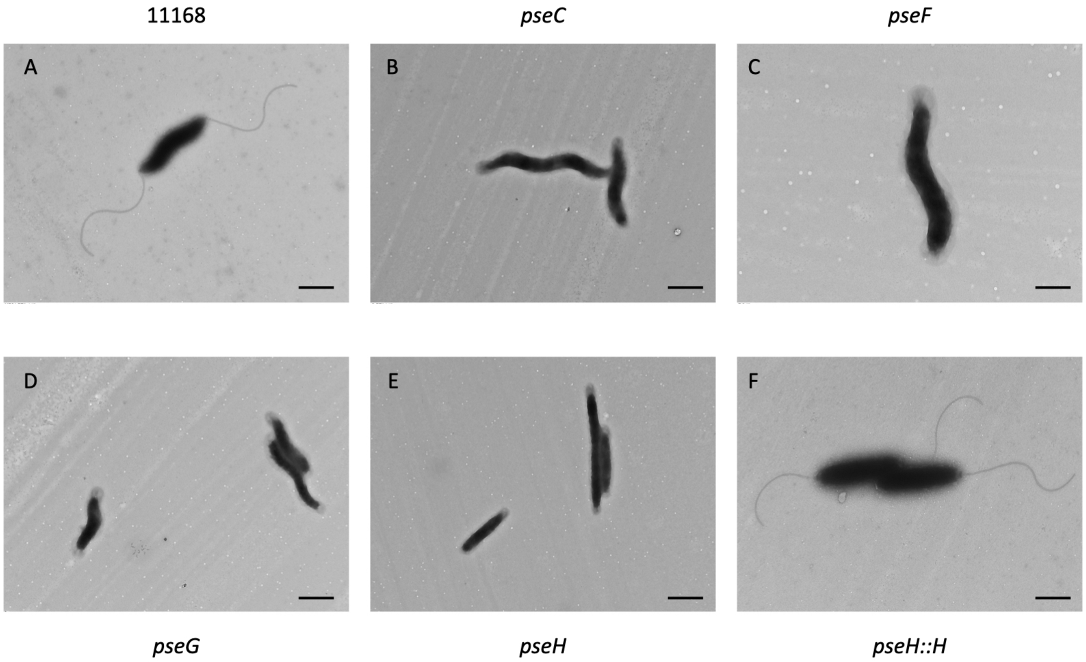

Figure S1. Transmission electron micrographs showing presence of flagella in wild type *C. jejuni* 11168 cells (**A**), absence of flagella in *pseC-H* mutants (**B–E**), and restoration of flagella in the *pseH*::*H* complement strain (**F**). Scale bars are 800 nm.

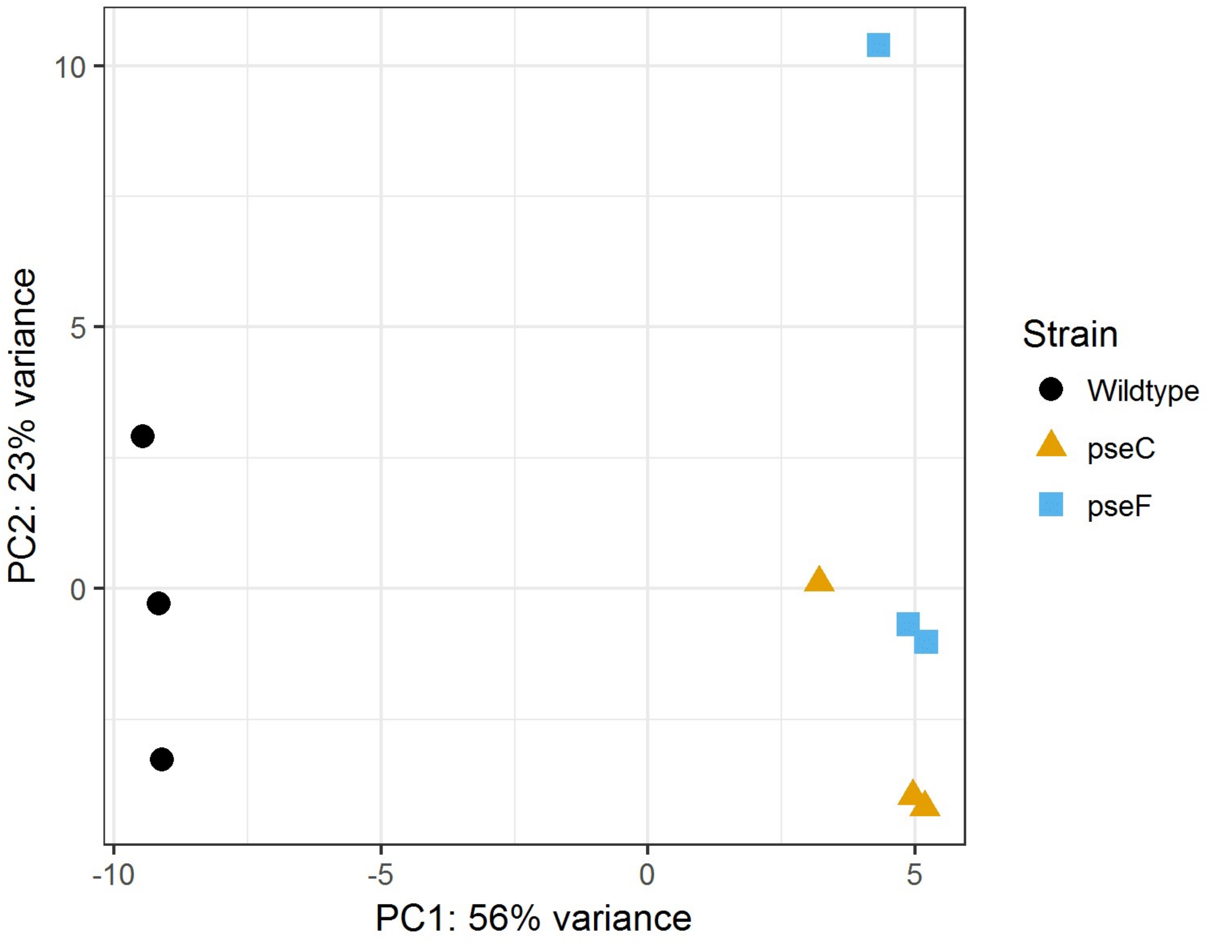

Figure S2. Principal component analysis (PCA) plot for *C. jejuni* NCTC 11168 wild type and mutant cells: wild type, *ΔpseC* and *ΔpseF*. Plots were generated using the data from the 500 genes with the greatest variation in expression across samples.

Table S1. Differentially expressed genes for *C. jejuni* NCTC 11168 wild type and mutant cells: *ΔpseC* vs. wild type, *ΔpseF* vs. wild type, and *ΔpseF* vs. *ΔpseC*. Genes with false discovery rate (FDR)-corrected *P*-value of < 0.05 (adjusted *P*-value, or padj) are shown. Changes are expressed as log_2foldChange, and are listed from lowest to highest adjusted *P*-value.

	***pseC* vs. wild type**	
**Gene Names**	**log_2 Fold Change**	**padj**
flgE2	-5.65	0.00
flaB	-4.69	0.00
metB	4.35	0.00
metA	4.34	0.00
cj0501	-4.33	0.00
flgE	-4.92	0.00
flgD	-4.67	0.00
cj0887c	-3.82	0.00
flgK	-3.77	0.00
flgH	-4.32	0.00
flgI	-4.37	0.00
gltB	-2.74	0.00
glnA	-3.71	0.00
flgG2	-3.44	0.00
cj0008	-2.71	0.00
flgG	-2.64	0.00
asd	-2.89	0.00
gltD	-2.60	0.00
fliK	-4.37	0.00
cj0391c	-1.98	0.00
cj1650	-4.36	0.00
cj1295	-5.47	0.00
cj1656c	-1.73	0.00
cj1242	-5.52	0.00
cj1022c	-1.76	0.00
cj0044c	-2.26	0.00
cj0040	-5.94	0.00

flgJ	-4.21	0.00
neuB2	-1.75	0.00
flgB	-1.97	0.00
cj1325	-1.54	0.00
flgR	-1.58	0.00
cj0977	-1.88	0.00
cj0859c	-2.03	0.00
flaA	-1.35	0.00
cj1465	-1.86	0.00
cj0243c	-4.25	0.00
hddA	1.22	0.00
fliD	-1.18	0.00
cj1422c	1.72	0.00
cj0554	-1.02	0.00
cj1296	-3.33	0.00
cj0552	-1.14	0.00
cj0553	-0.92	0.00
cj0986c	-1.48	0.00
cj1450	-1.14	0.00
rplN	1.07	0.00
rpsN	1.17	0.00
pseB	-0.93	0.00
livJ	-0.92	0.00
rplX	1.09	0.00
nrdA	0.88	0.00
acs	-1.12	0.00
rplP	0.99	0.00
putA	-0.77	0.00
rplB	0.91	0.00
purB	0.78	0.00
cj1631c	-1.22	0.00
rpsB	0.84	0.00
rplD	0.86	0.00
cj1500	1.27	0.00

rplW	1.02	0.00
flgM	-1.09	0.00
cj0021c	-0.75	0.00
rpsQ	0.99	0.00
neuC2	-1.24	0.00
rpsC	0.97	0.00
rpsH	0.78	0.00
fliS	-0.99	0.00
rpsS	0.92	0.00
secY	0.78	0.00
cj0045c	-1.50	0.00
cj0037c	-1.00	0.00
cj1020c	-1.01	0.00
glmS	0.70	0.00
pseC	-0.77	0.00
peb1A	-0.81	0.00
metK	0.72	0.00
cj1164c	0.73	0.00
rpmC	1.15	0.00
fusA	0.81	0.00
livK	-0.71	0.00
petB	0.65	0.00
peb3	0.72	0.00
rplV	0.89	0.00
pseD	-0.99	0.00
cj0920c	-0.62	0.00
cj0919c	-0.64	0.00
putP	-0.63	0.00
cj0089	0.67	0.00
cj0949c	0.89	0.00
kgtP	-0.56	0.00
rpsM	0.89	0.00
rpsL	0.86	0.00
cj1608	-0.86	0.00

cj1026c	-0.79	0.00
cj0203	-0.73	0.00
cj0935c	-0.67	0.00
pebC	-0.59	0.00
ilvE	0.67	0.00
rplO	0.83	0.00
petA	0.67	0.00
infC	0.70	0.00
rpoC	0.64	0.00
cj0989	-1.16	0.00
purL	0.75	0.00
cj1609	-0.60	0.00
cj1709c	1.23	0.00
cj1365c	0.64	0.00
ptmA	-0.85	0.00
thrS	0.56	0.00
rplR	0.79	0.00
rpmJ	0.99	0.00
cj0958c	0.65	0.00
rplE	0.82	0.00
argC	0.82	0.00
rplF	0.71	0.00
gmhA2	0.72	0.00
cj0947c	0.74	0.00
rpsE	0.77	0.01
purH	0.74	0.01
ppi	0.81	0.01
cj0091	0.67	0.01
dnaK	0.63	0.01
tsf	0.63	0.01
fdhC	-0.84	0.01
rpoB	0.52	0.01
rpmH	0.89	0.01
flaG	-0.76	0.01

rplL	0.71	0.01
rpsD	0.73	0.01
dut	-0.60	0.01
ung	-0.74	0.01
cj0550	-1.11	0.01
cj0903c	-0.67	0.01
cj0830	-0.68	0.01
cj0909	-0.56	0.01
cj1013c	-0.53	0.01
pstS	0.93	0.01
panD	0.65	0.01
rplQ	0.64	0.01
cj1426c	0.58	0.01
ilvC	0.59	0.02
cj0093	0.63	0.02
cj1034c	-0.88	0.02
cj1172c	0.77	0.02
rplM	0.69	0.02
fmt	0.89	0.02
cj0268c	0.60	0.02
rpmA	0.54	0.02
cj0505c	0.71	0.02
petC	0.49	0.02
fdhB	-0.77	0.02
cj1180c	1.00	0.02
mrp	0.52	0.02
p19	-0.74	0.02
cj0777	0.74	0.02
omp50	0.61	0.02
cj1191c	0.76	0.02
cj0073c	-0.48	0.02
cj0092	0.58	0.02
dapA	-0.68	0.02
rpsG	0.58	0.02

cj1342c	-0.69	0.02
cj0129c	0.49	0.02
cj1329	-0.67	0.03
ptmB	-0.62	0.03
htpG	0.49	0.03
rpoA	0.63	0.03
cydA	0.63	0.03
cj1340c	-0.75	0.03
pta	-0.46	0.03
hisH	-0.92	0.03
livM	-0.67	0.03
cj1075	-0.51	0.03
atpC	0.54	0.03
cj0417	-1.20	0.03
rpsP	0.70	0.04
cj0457c	-0.63	0.04
cj1666c	0.59	0.04
typA	0.51	0.04
cj1021c	-1.15	0.04
rpsK	0.64	0.04
panB	0.49	0.04
nusG	0.51	0.04
dnaJ	0.63	0.04
argD	0.97	0.05
dapB	0.49	0.05

	pseF **vs. wild type**	
**Gene Names**	**$\log_2$ Fold Change**	**padj**
flgE2	-7.73	0.00
flaB	-5.91	0.00
cj0501	-4.78	0.00
flgE	-6.15	0.00

flgK	-4.91	0.00
flgD	-6.42	0.00
flgI	-4.91	0.00
flgH	-4.73	0.00
glnA	-4.13	0.00
cj0887c	-3.41	0.00
gltB	-2.93	0.00
flgG2	-3.77	0.00
cj0008	-2.83	0.00
flgG	-2.98	0.00
gltD	-2.97	0.00
cj0391c	-2.36	0.00
fliK	-5.10	0.00
pseG	-4.91	0.00
cj1650	-4.82	0.00
cj1656c	-1.93	0.00
cj1242	-6.27	0.00
fliD	-1.72	0.00
flaA	-1.67	0.00
flgB	-2.01	0.00
cj0859c	-2.29	0.00
cj0977	-1.97	0.00
cj0044c	-2.07	0.00
flgJ	-5.76	0.00
cj0040	-6.57	0.00
acs	-1.55	0.00
cj1450	-1.39	0.00
pseB	-1.14	0.00
fliS	-1.51	0.00
cj1465	-1.90	0.00
pseF	-1.50	0.00
flaG	-1.45	0.00
nrdA	1.04	0.00
putA	-0.95	0.00

cj0243c	-4.02	0.00
peb1A	-1.16	0.00
cj1026c	-1.25	0.00
pseH	-4.99	0.00
fldA	-1.05	0.00
putP	-0.92	0.00
cj0554	-0.93	0.00
cj0553	-0.86	0.00
cj0037c	-1.31	0.00
cj1631c	-1.46	0.00
rpsN	1.13	0.00
cj0986c	-1.37	0.00
cj0552	-1.02	0.00
purB	0.84	0.00
cj0045c	-1.89	0.00
flgM	-1.19	0.00
cj0021c	-0.81	0.00
acnB	-0.96	0.00
pebC	-0.77	0.00
secY	0.83	0.00
cj0903c	-0.96	0.00
pseC	-0.84	0.00
mdh	-0.78	0.00
icd	-0.74	0.00
rplN	0.84	0.00
murD	1.02	0.00
sucC	-0.86	0.00
cj1500	1.18	0.00
cj0203	-0.85	0.00
sucD	-0.77	0.00
ribA	-0.86	0.00
cj1309c	-0.75	0.00
cj0949c	0.96	0.00
oorA	-0.68	0.00

rpsQ	0.90	0.00
oorD	-0.79	0.00
fdhA	-0.78	0.00
cj0073c	-0.67	0.00
cj0920c	-0.63	0.00
lctP	-0.73	0.00
dut	-0.74	0.00
cj1365c	0.70	0.00
cj0878	1.02	0.00
cj0864	-0.99	0.00
sdaA	-0.60	0.00
cj0415	-0.80	0.00
cj0834c	-0.65	0.00
rplX	0.79	0.00
oorC	-0.63	0.00
cj0604	-0.73	0.00
rplB	0.71	0.00
kgtP	-0.54	0.00
frdA	-0.70	0.00
cj1013c	0.61	0.00
cj0958c	0.68	0.00
cj0947c	0.79	0.00
oorB	-0.68	0.00
argC	0.85	0.00
cj0075c	-0.72	0.00
cj0833c	-0.57	0.00
cj0074c	-0.66	0.00
gltA	-0.68	0.00
ffh	0.68	0.00
cj1191c	0.92	0.00
clpB	-0.70	0.00
cj0777	0.88	0.00
livH	0.74	0.00
cj0873c	-0.77	0.00

lon	0.63	0.00
argD	1.25	0.01
rpsC	0.75	0.01
cj1180c	1.14	0.01
rplP	0.67	0.01
cj1215	0.69	0.01
cj0711	1.05	0.01
ksgA	0.86	0.01
flaC	-0.82	0.01
glmS	0.56	0.01
hisH	0.75	0.01
cj0716	-0.61	0.01
dnaX	0.66	0.01
cj0186c	0.86	0.01
cstA	-0.69	0.01
petB	0.53	0.01
fdhB	-0.84	0.01
rpsH	0.59	0.01
hydB	-0.56	0.01
cj1089c	0.98	0.01
cj0544	1.15	0.01
groEL	-0.65	0.01
cj1367c	0.74	0.01
cj0505c	0.76	0.01
cj0715	-0.63	0.01
ruvB	0.62	0.01
infC	0.63	0.01
rpsS	0.68	0.01
cj1609	-0.54	0.01
cj0559	-0.55	0.01
cj0826	0.92	0.01
cj0948c	1.04	0.01
cj0504c	1.10	0.01
cj0417	-1.44	0.01

fdhC	-0.79	0.01
cj1417c	0.93	0.01
thiC	-0.64	0.01
pseA	-0.63	0.01
cadF	-0.54	0.01
rplR	0.70	0.01
frdC	-0.51	0.01
selB	0.83	0.01
cj0485	-0.89	0.01
ispH	0.73	0.01
cj1074c	0.59	0.01
rpmC	0.88	0.01
p19	-0.79	0.01
cj0005c	-0.70	0.02
cj1501	1.46	0.02
cetA	-0.51	0.02
cj0448c	-0.56	0.02
cj0129c	0.51	0.02
pabB	1.08	0.02
cj0605	-0.57	0.02
argB	1.41	0.02
ppi	0.72	0.02
cj1373	0.86	0.02
cj1485c	1.13	0.02
cj0520	1.04	0.02
cj0154c	1.24	0.02
tsf	0.56	0.02
cj0264c	-0.62	0.02
cj0919c	-0.49	0.02
livM	0.68	0.02
cj1666c	0.64	0.02
cj0621	0.72	0.02
cj1154c	1.79	0.02
fmt	0.87	0.02

cj1476c	-0.50	0.02
cj0957c	0.70	0.03
mraY	0.62	0.03
dapD	0.61	0.03
purL	0.60	0.03
cj0122	-1.85	0.03
thrS	0.45	0.03
rplK	0.45	0.03
cj0489	-0.71	0.03
trmE	0.75	0.03
hypC	-0.66	0.03
mqo	-0.45	0.03
dcd	-0.63	0.03
cj0669	0.60	0.03
livG	0.83	0.03
ktrB	0.90	0.03
hisI	0.66	0.03
serS	-0.50	0.03
cj1164c	0.50	0.03
rpoN	0.66	0.03
cj0256	0.77	0.03
peb3	0.50	0.03
serA	-0.48	0.03
cj1602	0.68	0.03
cj0935c	-0.51	0.03
uxaA'	-0.67	0.03
cj0830	-0.62	0.03
gmhA	-0.54	0.03
flgC	-0.52	0.03
hydA	-0.56	0.03
rplF	0.57	0.03
cj0353c	0.64	0.03
gatC	-0.60	0.03
hemB	-0.52	0.03

nuoI	-0.50	0.03
glmM	-0.55	0.03
cj0089	0.48	0.03
cydA	0.61	0.03
maf	0.95	0.03
cj1324	-0.44	0.03
typA	-0.51	0.04
ilvI	-0.47	0.04
hisF	0.65	0.04
cj1541	-0.48	0.04
rpsL	0.62	0.04
nuoB	-0.56	0.04
cj0573	-0.64	0.04
nuoD	-0.43	0.04
cj0592c	-0.61	0.04
murF	-0.50	0.04
rplW	0.59	0.04
sdhC	-0.46	0.04
cj0550	-0.94	0.04
trxB	-0.51	0.04
cmeF	0.53	0.04
purD	-0.46	0.04
cmeA	-0.75	0.04
cj0440c	-0.63	0.04
cj0832c	-0.41	0.04
dsbB	-0.72	0.04
metE	-0.82	0.04
ispDF	-0.45	0.05
rpsO	0.71	0.05
rpsB	0.47	0.05
cj0608	0.61	0.05

	pseF vs. *pseC*	
Gene Names	**$\log_2$ Fold Change**	**padj**
metB	-4.81	0.00
metA	-4.77	0.00
pseG	-4.84	0.00
asd	2.43	0.00
cj1295	5.00	0.00
cj1022c	1.78	0.00
cj1422c	-2.49	0.00
flgE2	-2.08	0.00
livJ	1.28	0.00
cj1325	1.39	0.00
neuB2	1.48	0.00
flgR	1.41	0.00
cj1013c	1.14	0.00
livH	1.24	0.00
livM	1.34	0.00
livK	0.99	0.00
pseH	-4.73	0.00
typA	-1.02	0.00
cj1020c	1.28	0.00
hddA	-1.00	0.00
flaB	-1.22	0.00
neuC2	1.33	0.00
cj1296	2.75	0.00
flgD	-1.75	0.00
cj1426c	-0.84	0.00
livG	1.28	0.00
pseF	-1.01	0.00
livF	1.11	0.00
flgK	-1.14	0.00
cj1021c	1.60	0.00
cj1012c	1.83	0.00
gmhA2	-0.79	0.00

cj1330	0.98	0.01
flgE	-1.23	0.01
cj1075	0.65	0.01
cj1329	0.81	0.01
fldA	-0.64	0.01
rpsI	-0.66	0.02
frdA	-0.63	0.02
cj0669	0.68	0.03
cj1309c	-0.58	0.03
rpoN	0.74	0.03
groEL	-0.63	0.03
cj0989	1.01	0.03
sdhC	-0.55	0.04

Table S2. Complete list of genetic polymorphisms and variant frequencies between NCTC 12673 phage (parent) and MutC, a spontaneous mutant phage isolated on *C. jejuni* 11168 *ΔpseC* mutant cells.

Gene	Predicted function	Phage	Min	Max	Amino Acid Change	Change	Codon Change	Polymorphism Type	Variant Frequency
gp047	FlaGrab, flagellar glycan binding protein	MutC	40,821	40,821	Frame Shift	(A)7 -> (A)6	Frame Shift	Deletion (tandem repeat)	68.80%
gp114/ gp115	Hypothetical protein	MutC	100,737	100,736	Frame Shift	(CC)4 -> (CC)5	Frame Shift	Insertion (tandem repeat)	37.00%
gp116	Hypothetical protein	Parent	101,692	101,691	Frame Shift	(C)11 -> (C)12	Frame Shift	Insertion (tandem repeat)	28.90%
gp116	Hypothetical protein	Parent	101,691	101,691	Frame Shift	(C)11 -> (C)10	Frame Shift	Deletion (tandem repeat)	27.10%
gp116	Hypothetical protein	MutC	101,691	101,691	Frame Shift	(C)11 -> (C)10	Frame Shift	Deletion (tandem repeat)	62.80%

gp041	gp6 baseplate wedge subunit	MutC	31,844	31,844	L -> S	A -> G	TTA -> TCA	SNP (transition)	72.70%
gp058	Hef59	MutC	50,606	50,607	E -> G	AA -> GG	GAA -> GGG	Substitution	69.8% - 69.9%
gp167	Hef168	MutC	132,316	132,316	D -> N	C -> T	GAT -> AAT	SNP (transition)	64.00%
gp116	Hypothetical protein	MutC	101,202	101,202	T -> M	G -> A	ACG -> ATG	SNP (transition)	68.70%
gp116	Hypothetical protein	Parent	101,112	101,112	T -> N	G -> T	ACT -> AAT	SNP (transversion)	73.80%
gp041	gp6, baseplate wedge subunit	MutC	31,885	31,885	M -> I	C -> T	ATG -> ATA	SNP (transition)	25.60%
gp114/ gp115	Hypothetical protein	Parent	100,737	100,736	Frame Shift	(C)9 -> (C)10	Frame Shift	Insertion (tandem repeat)	31.20%
gp114/ gp115	Hypothetical protein	MutC	100,737	100,736	Frame Shift	(C)9 -> (C)10	Frame Shift	Insertion (tandem repeat)	43.70%
up-stream of *gp113*	Intergenic region	Parent	99,920	99,921	N/A (not within a CDS)	AC -> GT	N/A (not within a CDS)	Substitution	73.9% - 77.1%
up-stream of *gp113*	Intergenic region	MutC	99,920	99,921	N/A (not within a CDS)	AC -> GT	N/A (not within a CDS)	Substitution	98.5% - 98.6%
gp047	Putative tail fibre	Parent	40,935	40,935	Frame Shift	(A)7 -> (A)6	Frame Shift	Deletion (tandem repeat)	33.20%
gp047	Putative tail fibre	MutC	40,935	40,935	Frame Shift	(A)7 -> (A)6	Frame Shift	Deletion (tandem repeat)	25.30%
